# Complications following immediate compared to delayed deep inferior epigastric artery perforator flap breast reconstructions

**DOI:** 10.1007/s10549-018-4695-0

**Published:** 2018-02-05

**Authors:** J. Beugels, L. Bod, S. M. J. van Kuijk, S. S. Qiu, S. M. H. Tuinder, E. M. Heuts, A. Piatkowski, R. R. W. J. van der Hulst

**Affiliations:** 1 Department of Plastic, Reconstructive and Hand Surgery, Maastricht University Medical Centre, P.O. Box 5800, 6202 AZ Maastricht, The Netherlands; 2 GROW – School for Oncology and Developmental Biology, Maastricht University Medical Center, Maastricht, The Netherlands; 3 Department of Surgery, Maastricht University Medical Center, Maastricht, The Netherlands; 4 Department of Clinical Epidemiology and Medical Technology Assessment, Maastricht University Medical Center, Maastricht, The Netherlands; 5 Department of Plastic, Reconstructive and Hand Surgery, VieCuri Medical Center, Venlo, The Netherlands; 6 Department of Plastic, Reconstructive and Hand Surgery, Zuyderland Medical Center, Sittard-Geleen, The Netherlands

**Keywords:** Breast reconstruction, DIEP flap, Perforator flap, Microsurgery, Complications

## Abstract

**Purpose:**

As more breast cancer patients opt for immediate breast reconstruction, the incidence of complications should be evaluated. The aim of this study was to analyze the recipient-site complications and flap re-explorations of immediate compared to delayed deep inferior epigastric artery perforator (DIEP) flap breast reconstructions.

**Methods:**

For this multicenter retrospective cohort study, the medical records of all patients who underwent DIEP flap breast reconstruction in three hospitals in the Netherlands between January 2010 and June 2017 were reviewed. Patient demographics, risk factors, timing of reconstruction, recipient-site complications, and flap re-explorations were recorded.

**Results:**

A total of 910 DIEP flap breast reconstructions (*n* = 397 immediate and *n* = 513 delayed reconstructions) in 737 patients were included. There were no significant differences in major complications or flap re-explorations between immediate and delayed reconstructions. The total flap failure rate was 1.5 and 2.5% in the immediate and delayed group, respectively. Significantly more hematomas (OR 2.91; 95% CI 1.59–5.30; *p* = 0.001) and seromas (OR 3.60; 95% CI 1.14–11.4; *p* = 0.029) occurred in immediate reconstructions, whereas wound problems were more frequently observed in delayed reconstructions (OR 1.99; 95% CI 1.27–3.11; *p* = 0.003). Correction for potential confounders still showed significant differences for hematoma and seroma, but no longer for wound problems (*p* = 0.052).

**Conclusions:**

This study demonstrated similar incidences of major recipient-site complications and flap re-explorations between immediate and delayed DIEP flap breast reconstructions. However, hematoma and seroma occurred significantly more often in immediate reconstructions, while wound problems were more frequently observed in delayed reconstructions.

**Electronic supplementary material:**

The online version of this article (10.1007/s10549-018-4695-0) contains supplementary material, which is available to authorized users.

## Purpose

Over the last two decades, the number of women seeking breast reconstruction has increased. Even though the main indication for mastectomy remains breast cancer, there has been in increase in prophylactic, risk-reducing mastectomy as a result of the advancements in genetic testing and the general overestimation of the risk of contralateral breast cancer [1–10]. In addition, studies have shown that younger patients and patients undergoing bilateral mastectomy are more likely to opt for breast reconstruction [6, 7, 11]. Currently there are several reconstruction options available using either implants, autologous tissue, or a combination of both.

The reconstruction technique that is preferred is influenced by multiple factors including tissue availability, indications for adjuvant therapy, reimbursement issues, institutional factors like hospital setting (university or community hospital), operating room availability, and the expertise of the reconstructive surgeons [7, 12–14]. Even though a paradigm shift away from autologous tissue to implant-based techniques has been observed in the United States [6–8, 11], numerous studies have indicated that autologous reconstructions provide a more natural and permanent result with higher patient satisfaction rates [14–16]. Out of all options for autologous breast reconstruction, the deep inferior epigastric artery perforator (DIEP) flap is the first choice in most centers [17, 18].

An important part of the decision-making process of breast reconstruction is selecting the appropriate timing of the reconstruction. Breast reconstruction can be performed either immediately in the same operation as the mastectomy or in a delayed setting months to years after mastectomy. An important factor that influences the timing of the reconstruction is having an indication for post-mastectomy radiation therapy (PMRT). Although PMRT has been associated with increased complications and poorer outcomes in immediate reconstructions, the literature regarding the optimal timing of radiation therapy and breast reconstruction remains controversial [19–22]. When PMRT is not considered, immediate reconstructions yield better aesthetic results, are more cost-effective, and may protect breast cancer patients from a period of psychological distress, poor body image, and diminished sexual well-being compared to delayed breast reconstructions [23, 24].

However, the availability of immediate breast reconstruction and the number of immediate autologous breast reconstructions performed is highly variable among institutions [25, 26]. As more women opt for immediate breast reconstruction, the incidence of complications following immediate and delayed autologous breast reconstruction should be evaluated. Large series that compare the overall complications after immediate versus delayed DIEP flap breast reconstructions are lacking. Most studies that evaluated the complication rates compared autologous to implant-based reconstructions and focused on the role of PMRT. The aim of this study was to analyze the recipient-site complications and flap re-explorations of immediate compared to delayed DIEP flap breast reconstructions.

## Methods

A multicenter retrospective cohort was constructed from a prospectively maintained database of all patients who underwent DIEP flap breast reconstruction at Maastricht University Medical Center in the Netherlands and two community hospitals, VieCuri Medical Center, Venlo, and Zuyderland Medical Center, Sittard-Geleen, between January 2010 and June 2017. The study was approved by the medical ethics committee and was performed in accordance with the ethical standards of the Declaration of Helsinki.

Medical records were reviewed and data that were collected included patient demographics and risk factors, timing of reconstruction (immediate or delayed), type of reconstruction (unilateral or bilateral), operative details, recipient-site complications, and re-explorations of the flap. The follow-up length was quantified as the time between the date of operation and the last visit to the outpatient clinic.

The vast majority of bilateral breast reconstructions were performed in the university hospital, while unilateral reconstructions were performed in all three centers. All consecutive patients who underwent DIEP flap breast reconstruction within the study period were considered for inclusion. However, patients with stacked unilateral or mixed bilateral (immediate on one side and delayed on the other side) DIEP flap breast reconstructions were excluded.

In the Netherlands, breast reconstruction is integrated in the total breast cancer care. The plastic surgeons closely collaborated with the breast surgeons to counsel all patients appropriately. Patients who were oncologically eligible could opt for immediate breast reconstruction. If patients had a preoperative indication or had a high chance for PMRT, delayed DIEP flap breast reconstruction was advised. Another frequent reason for delayed DIEP flap breast reconstruction was the problems related to implants. Patients with a genetic predisposition (i.e., BRCA1, BRCA2, and CHEK2 mutation carriers) underwent prophylactic mastectomies followed by immediate breast reconstruction. Skin-sparing mastectomies were performed in all cases of immediate breast reconstruction.

The preoperative work-up was standard for all patients and included a physical examination, preoperative markings of the flap, and localization of the deep inferior epigastric artery perforators with a hand-held Doppler device. Preoperative imaging with magnetic resonance angiography (MRA) of the abdomen was performed in all patients scheduled for DIEP flap breast reconstruction in the university hospital. The internal mammary vessels served as the recipient vessels in all cases. Flaps were monitored by checking the Doppler signals, color, and capillary refill every hour for the first 24 h, every 2 h on the second day, every 4 h on the third day, every 8 h on the fourth day, and once daily on each additional day if the hospital stay was more than four days. All patients received antibiotics intra- and postoperatively. The patients also received prophylactic low-molecular-weight heparin until discharge and wore pressure stockings during the operation and the time of immobilization.

The primary outcome was the incidence of major and minor recipient-site complications. Major complications included total flap loss, partial flap loss, and venous congestion of the flap. Infection, hematoma, seroma, fat necrosis, and wound problems were considered minor complications. Wound problems included wound dehiscence and superficial skin necrosis related to the breast reconstruction, but not necrosis of mastectomy skin. Fat necrosis was defined as a palpable firmness identified by physical examination during postoperative evaluation or detected by ultrasound. Cases of partial flap loss were also registered as fat necrosis. The secondary outcome was the need for re-explorations of the flap. The reason for re-exploration and the final result were evaluated.

### Data Analysis

Continuous variables were presented as mean and standard deviation or as median and interquartile range (IQR) depending on the distribution of the data. Categorical variables were presented as absolute numbers and percentages. Continuous outcome variables were compared with the independent samples *t* test or the Mann–Whitney *U* test as appropriate. Categorical data were tested with a Chi-square or Fisher’s exact test. The primary unit of analysis was the flap—rather than the patient—for complication analyses. Even though in a bilateral breast reconstruction both flaps are harvested from the same abdomen and are therefore not independent, complications can occur for each flap separately. Generalized estimating equations (GEE) was used to compute the association between immediate or delayed breast reconstruction with all major and minor recipient-site complications. This method ensures correction for the fact that some patients underwent bilateral breast reconstruction and thus provide clustered data. The use of GEE to account for clustered data was defined a priori and was not based on the statistical comparison of models. In addition to these univariate analyses, the odds ratios were corrected for potential confounding variables (i.e., unilateral vs. bilateral reconstruction, university vs. community hospital, body mass index, smoking status, radiation therapy, chemotherapy, and endocrine therapy) in multivariable models. Subgroup analyses were done for patients who had breast cancer, thereby excluding reconstructions after prophylactic mastectomy. All *p* values ≤ 0.05 were considered statistically significant. Statistical analyses were conducted using IBM SPSS (version 23.0, SPSS Inc., Chicago, Illinois, USA) for Windows.

## Results

### Patient demographics

Between January 2010 and June 2017, a total of 1012 DIEP flap breast reconstructions were performed in 788 patients. After excluding stacked unilateral and mixed bilateral reconstructions, 910 DIEP flap breast reconstructions (*n* = 397 immediate and *n* = 513 delayed reconstructions) in 737 patients were included. In Maastricht University Medical Center, 577 flaps (63.4%; *n* = 299 immediate and *n* = 278 delayed) were performed in 406 patients; in VieCuri Medical Center, 165 flaps (18.1%; *n* = 92 immediate and *n* = 73 delayed) were performed in 165 patients; and in Zuyderland Medical Center, 168 flaps (18.5%; *n* = 6 immediate and *n* = 162 delayed) were performed in 166 patients. Median follow-up was 9 months (IQR 4–19 months) and 10 months (IQR 4–18 months) in the immediate and delayed reconstruction group, respectively. The patient demographics are presented in Table 1.

**Table 1:** Patient demographics (*n* = 737 patients)

	Immediate reconstruction *n* (%)	Delayed reconstruction *n* (%)	*p* value
Total number of patients	291	446	
Total number of DIEP flaps	397	513	
Age in years; mean ± SD	50.7 ± 9.4	51.0 ± 8.6	0.657
BMI; mean ± SD	26.6 ± 3.7	27.1 ± 3.8	0.064
Active smoker	24 (8.2)	50 (11.2)	0.191
Hypertension	49 (16.8)	77 (17.3)	0.881
Diabetes Mellitus	14 (4.8)	20 (4.5)	0.836
Genetic predisposition	68 (23.4)	23 (5.2)	< 0.001
History of lumpectomy^a^	75 (18.9)	70 (13.6)	0.032
History of tissue expanders/implants^a^	5 (1.3)	162 (31.6)	< 0.001
Type of reconstruction			
Unilateral	185 (63.6)	379 (85.0)	< 0.001
Bilateral	106 (36.4)	67 (15.0)	
Reason for mastectomy^a^			
Oncological	243 (61.2)	453 (88.3)	< 0.001
Risk-reducing or prophylactic	154 (38.8)	60 (11.7)	
Oncological treatment			
History of radiation therapy^a^	86 (21.7)	228 (44.4)	< 0.001
Radiation therapy on DIEP flap^a^	22 (5.5)	0 (0)	< 0.001
Chemotherapy	119 (40.9)	296 (66.4)	< 0.001
Endocrine therapy	100 (34.4)	235 (52.7)	< 0.001
Immunotherapy	34 (11.7)	59 (13.2)	0.537
Hospital setting			
University hospital	193 (66.3)	213 (47.8)	< 0.001
Community hospital	98 (33.7)	233 (52.2)	
Follow-up in months; median (IQR)	9 (4–19)	10 (4–18)	0.975

*DIEP* deep inferior epigastric artery perforator, *SD* standard deviation, *BMI* body mass index, *IQR* interquartile range

^a^Total number of flaps as unit of analysis (immediate group: *n* = 397; delayed group: *n* = 513)

Patients in the immediate reconstruction group significantly more often had a genetic predisposition to breast cancer (i.e., BRCA1, BRCA2, and/or CHEK2 mutations; *p* < 0.001) and a lumpectomy in their medical history (*p* = 0.032). Furthermore, they underwent prophylactic mastectomies and bilateral DIEP flap breast reconstructions (*p* < 0.001 for both) more frequently compared to the delayed group. On the other hand, patients in the delayed group more often had a history of tissue expanders or implants (*p* < 0.001) and more frequently underwent mastectomy due to breast cancer followed by unilateral DIEP flap reconstructions (*p* < 0.001). Therefore, significantly more patients in the delayed reconstruction group had a history of radiation therapy, chemotherapy, and endocrine therapy.

### Operative details

An overview of the operative details is provided in Table 2. The median operative time in minutes was significantly less in delayed unilateral breast reconstructions compared to immediate unilateral reconstruction (352 min vs. 380 min; *p* < 0.001), but there was no difference in operative time in bilateral breast reconstruction. Flaps were significantly larger in delayed breast reconstructions (*p* < 0.001). Flap weight and BMI were directly correlated (Pearson’s *r* = 0.642; *p* < 0.001). However, it should be noted that in the delayed group 85.0% were unilateral breast reconstructions compared to only 63.6% in the immediate group (*p* < 0.001).

**Table 2:** Operative details (*n* = 737 DIEP flap breast reconstruction procedures)

	Immediate reconstruction *n* (%)	Delayed reconstruction *n* (%)	*p* value
Total number of DIEP flap procedures	291	446	
Operative time in minutes; median (IQR)			
Unilateral breast reconstructions	380 (325–437)	352 (293–404)	< 0.001
Bilateral breast reconstructions	456 (390–534)	467 (404–548)	0.192
Ischemia time in minutes; median (IQR)^a^	47 (40–60)	50 (40–65)	0.123
Flap weight in grams; mean ± SD^a^	637 ± 233	729 ± 250	< 0.001
Hospital stay in days; median (IQR)	7 (6–7)	7 (6–7)	0.192

*DIEP* deep inferior epigastric artery perforator, *IQR* interquartile range, *SD* standard deviation

^a^Total number of flaps as unit of analysis (immediate group: *n* = 397; delayed group: *n* = 513)

### Recipient-site complications

Overall, major and minor recipient-site complications were observed in 9.6% (87/910 flaps) and 24.0% (218/910 flaps) of flaps, respectively, with no differences between groups. Specific recipient-site complications of all flaps are summarized in Table 3. No statistically significant differences in any of the major complications were observed between immediate and delayed DIEP flap breast reconstructions. The average total flap failure rate was 2.1% (19/910 flaps), whereas it was 1.5% and 2.5% in the immediate and delayed group, respectively.

**Table 3:** Recipient-site complications (*n* = 910 flaps)

	Timing of reconstruction		OR (95% CI)	*p* value	Adjusted OR (95% CI)^a^	Adjusted *p* value^a^
	Immediate (*n* = 397), *n* (%)	Delayed (*n* = 513), *n* (%)				
Major complication (≥ 1)	35 (8.8)	52 (10.1)	0.85 (0.54–1.34)	0.485	0.80 (0.49–1.31)	0.376
Total flap loss	6 (1.5)	13 (2.5)	0.60 (0.23–1.58)	0.300	0.49 (0.20–1.16)	0.106
Partial flap loss	14 (3.5)	25 (4.9)	0.72 (0.36–1.44)	0.357	0.74 (0.34–1.62)	0.455
Venous congestion	19 (4.8)	21 (4.1)	1.17 (0.61–2.23)	0.639	0.94 (0.49–1.82)	0.863
Minor complication (≥ 1)	93 (23.4)	125 (24.4)	0.95 (0.69–1.31)	0.735	1.18 (0.83–1.68)	0.360
Infection	25 (6.3)	38 (7.4)	0.84 (0.49–1.45)	0.529	1.06 (0.58–1.95)	0.853
Hematoma	36 (9.1)	17 (3.3)	2.91 (1.59–5.30)	0.001	3.22 (1.68–6.18)	< 0.001
Seroma	11 (2.8)	4 (0.8)	3.60 (1.14–11.4)	0.029	5.28 (1.36–20.5)	0.016
Fat necrosis	37 (9.3)	64 (12.5)	0.72 (0.47–1.11)	0.137	0.87 (0.55–1.38)	0.550
Wound problems	35 (8.8)	80 (15.6)	0.50 (0.32–0.79)	0.003	0.63 (0.40–1.00)	0.052

^a^Adjusted for unilateral vs. bilateral reconstruction, body mass index (kg/m^2^), smoking status (yes vs. no), radiation therapy (yes vs. no), chemotherapy (yes vs. no), endocrine therapy (yes vs. no), and university vs. community hospital

Even though the probability of having one or more minor complications was not significantly different between groups, the incidences of hematoma, seroma, and wound problems differed significantly between immediate and delayed DIEP flap breast reconstructions. Significantly more hematomas (OR 2.91; 95% CI 1.59–5.30; *p* = 0.001) and seromas (OR 3.60; 95% CI 1.14–11.4; *p* = 0.029) were observed in immediate reconstructions. On the other hand, wound problems including wound dehiscence and superficial skin necrosis were more frequently observed in delayed DIEP flap breast reconstructions (OR 1.99; 95% CI 1.27–3.11; *p* = 0.003). The other minor complications did not significantly differ between both groups.

The odds ratios of the univariate analyses were adjusted for potential confounding variables (i.e., unilateral vs. bilateral reconstruction, university or community hospital, BMI, smoking status, radiation therapy, chemotherapy, and endocrine therapy) in multivariable models. However, the adjusted odds ratios of hematoma (OR 3.22; 95% CI 1.68–6.18; *p* ≤ 0.001) and seroma (OR 5.28; 95% CI 1.36–20.5; *p* = 0.016) still showed statistically significant associations with immediate and delayed reconstructions. Interestingly, the adjusted odds ratio of wound problems (OR 1.59; 95% CI 1.00–2.53; *p* = 0.052) was no longer statistically significant, indicating that at least part of the association was due to confounders instead of timing of breast reconstruction.

In addition, subgroup analysis was performed to analyze the incidences of major and minor recipient-site complications in breast cancer patients only (*n* = 243 immediate and *n* = 453 delayed reconstructions in 664 patients), thereby omitting breast reconstructions after prophylactic mastectomies (Table 4). Both univariate and multivariable analyses of this subgroup showed similar results to the total group, with significantly more hematomas and seromas in immediate reconstructions and more wound problems in delayed reconstructions. However, the odds ratio of wound problems (OR 1.91; 95% CI 1.11–3.29; *p* = 0.020) remained statistically significant after correction for potential confounders in this subgroup of patients.

**Table 4:** Subgroup analysis recipient-site complications of breast cancer patients only (*n* = 696 flaps)

	Timing of reconstruction		OR (95% CI)	*p* value	Adjusted OR (95% CI)^a^	Adjusted *p* value^a^
	Immediate (*n* = 243), *n* (%)	Delayed (*n* = 453), *n* (%)				
Major complication (≥ 1)	19 (7.8)	45 (9.9)	0.77 (0.44–1.35)	0.358	0.75 (0.40–1.38)	0.347
Total flap loss	2 (0.8)	11 (2.4)	0.33 (0.07–1.52)	0.155	0.30 (0.06–1.49)	0.139
Partial flap loss	8 (3.3)	22 (4.9)	0.67 (0.29–1.52)	0.336	0.61 (0.24–1.58)	0.311
Venous congestion	10 (4.1)	17 (3.8)	1.10 (0.50–2.44)	0.809	1.11 (0.49–2.49)	0.804
Minor complication (≥ 1)	62 (25.5)	112 (24.7)	1.02 (0.71–1.47)	0.911	1.18 (0.81–1.74)	0.391
Infection	18 (7.4)	35 (7.7)	0.95 (0.52–1.72)	0.858	1.12 (0.59–2.13)	0.739
Hematoma	26 (10.7)	14 (3.1)	3.76 (1.93–7.35)	< 0.001	3.68 (1.85–7.31)	< 0.001
Seroma	7 (2.9)	3 (0.7)	4.45 (1.14–17.4)	0.032	8.32 (2.06–33.6)	0.003
Fat necrosis	27 (11.1)	58 (12.8)	0.85 (0.52–1.38)	0.515	0.95 (0.57–1.57)	0.831
Wound problems	19 (7.8)	74 (16.3)	0.43 (0.25–0.73)	0.002	0.52 (0.30–0.91)	0.020

^a^Adjusted for unilateral versus bilateral reconstruction, body mass index (kg/m^2^), smoking status (yes vs. no), radiation therapy (yes vs. no), chemotherapy (yes vs. no), endocrine therapy (yes vs. no), and university vs. community hospital

Lastly, univariate and multivariable (subgroup) analyses of recipient-site complications with the patient as the unit of analysis showed comparable results (see additional data “Online Resource 1: Table 1 and 2”).

### Flap re-explorations

An overview of the flap re-explorations is presented in Table 5. Overall, 67 DIEP flaps (7.4%) required re-exploration and 40 flaps (4.4%) reanastomosis of the vein and/or artery. There were no statistically significant differences in the number of flap re-explorations or reanastomoses between immediate and delayed reconstructions. Venous congestion was the most frequent reason for re-exploration of the flap, followed by arterial insufficiency and hematoma. On average, 64.2% (43/67 cases) of the flap re-explorations resulted in a viable flap. Analysis of flap re-explorations with the patient as the unit of analysis also showed similar results (see additional data “Online Resource 1: Table 3”).

**Table 5:** Flap re-explorations (*n* = 910 flaps)

	Timing of reconstruction		*p* value
	Immediate (*n* = 397), *n* (%)	Delayed (*n* = 513), *n* (%)	
Re-exploration	29 (7.3)	38 (7.4)	0.941
Reanastomosis	20 (5.0)	20 (3.9)	0.426
Reason re-exploration			
Arterial insufficiency	4 (1.0)	12 (2.3)	0.130
Venous insufficiency	20 (5.0)	20 (3.9)	0.406
Hematoma	5 (1.3)	6 (1.2)	1.000^a^
Kinking	4 (1.0)	5 (1.0)	1.000^a^
Infection	0 (0)	1 (0.2)	1.000^a^
Other	1 (0.3)	2 (0.4)	1.000^a^
Result re-exploration^b^			
Viable flap	21 (72.4)	22 (57.8)	0.219
Partial flap loss	2 (6.9)	8 (21.1)	0.168^a^
Total flap loss	6 (20.7)	8 (21.1)	0.971

^a^Fisher’s Exact test was used

^b^As a percentage of the total flaps that required re-exploration (immediate group: *n* = 29; delayed group: *n* = 38)

## Discussion

The aim of this study was to evaluate the recipient-site complications and flap re-explorations of immediate compared to delayed DIEP flap breast reconstructions.

Our comprehensive review demonstrated comparable major complication and flap re-exploration rates for immediate and delayed DIEP flap breast reconstructions. Analysis of minor recipient-site complications, however, showed that hematoma and seroma occurred significantly more often in immediate reconstructions, whereas wound problems were more frequently observed in the delayed reconstruction group. Also after correction for potential confounders, significant differences were found for hematoma and seroma, but no longer for wound problems (*p* = 0.052).

This indicates that one or more of these potential confounders have a significant impact on the incidence of wound problems. A previous study by our group already pointed out that higher BMI is a risk factor that is significantly associated with recipient-site complications of DIEP flap breast reconstructions (multivariable analysis: OR 1.137 per 1 point increase in BMI; 95% CI 1.075–1.201; *p* < 0.001) [27]. The patients who underwent delayed DIEP flap breast reconstructions had a higher BMI (immediate 26.6 ± 3.7 kg/m^2^ and delayed 27.1 ± 3.8 kg/m^2^; *p* = 0.064) and larger flaps (immediate 637 ± 233 grams and delayed 729 ± 250 grams; *p* ≤ 0.001) than patients with immediate breast reconstructions which might at least partially explain the clinically relevant difference in wound problems in this study. This finding is in line with a study by Chang et al. that also reported that BMI was a significant risk factor for postoperative complications, specifically wound dehiscence, in free-flap breast reconstructions. Patients with a BMI greater than 30 kg/m^2^ were more than twice as likely to develop a complication than patients with a normal BMI [28]. Another study reported that a higher BMI predisposes patients to delayed wound healing complications of the recipient site [29]. Surprisingly, a recent study by Lam et al. stated that increased flap weight, which was directly correlated with BMI, was not associated with flap complications, but patients with flaps over 667.5 grams were more likely to have donor-site wound healing problems. However, only mastectomy skin flap necrosis and not specifically recipient-site wound healing problems of the flap were assessed [30].

Interestingly, the adjusted odds ratios of hematoma and seroma still showed statistically significant differences in immediate compared to delayed reconstructions. Because of the low number of events, it was not possible to analyze the associations between hematoma or seroma and specific risk factors properly. The differences might be explained by the fact that immediate breast reconstruction includes more extensive surgery of both breast and axilla at the time of breast reconstruction and consequently a higher risk of active bleeding and seroma formation. Only mastectomy skin flap necrosis could reliably be attributed to mastectomy surgery and was therefore excluded from complication analyses, while all hematomas and seromas were recorded even when located in the lateral part of the reconstructed breast near the axilla. Despite the incidence of these minor complications, the superior aesthetic outcome and less psychological distress as advantages of immediate DIEP flap breast reconstruction should also be considered [24]. Furthermore, it should be emphasized that it is important to double-check the total surgical field in order to achieve adequate hemostasis and prevent seroma formation in immediate breast reconstruction in particular, since the reconstructive surgeon has the final responsibility.

Additionally, subgroup analysis was conducted of the recipient-site complications of all patients who had mastectomy due to breast cancer (61.2% of the flaps in the immediate group and 88.3% in the delayed reconstruction group) to take the differences in baseline characteristics and risk factors into account. Patients who opt for prophylactic mastectomy generally have less comorbidities and have no indication for adjuvant therapy and were therefore omitted. Similar results to the total group were found, with significantly more hematomas and seromas in immediate reconstructions and more wound problems in delayed reconstructions even after correction for confounders.

Limitations of this study include the retrospective design and the potential bias as a result. Data are affected by the reliability of accurate record keeping by different surgeons in three institutions. Another factor that may have affected outcome data is that the university hospital, where the majority of breast reconstructions have been performed (577/910 flaps; 63.4%), is a tertiary referral center to which more complex cases are referred. The number of immediate reconstructions was not evenly distributed over the three hospitals, yet the outcomes were adjusted for hospital setting. Selection bias was limited because all patients, except stacked unilateral and mixed bilateral reconstructions, were included. Lastly, the low number of events limited analyzing potentially significant confounders for hematoma and seroma formation.

The strengths of this multicenter study, on the other hand, include the large sample size of immediate and delayed DIEP flap breast reconstructions and the comprehensive statistical analysis of the recipient-site complications. In our opinion, these multicenter data rather reflect the general situation more realistically than single institution or single-surgeon studies, which increases the generalizability of our results. The large series of DIEP flaps made multivariable analyses with correction for clinically relevant confounding variables possible. An important observation of this study is that per-patient analysis provided comparable results to the per-flap analysis which facilitates the interpretation and translation of these findings to the clinic.

## Conclusions

This study demonstrated similar incidences of major recipient-site complications, including flap loss and venous congestion, between immediate and delayed DIEP flap breast reconstructions. The rate of flap re-exploration was comparable in both groups. Analysis of minor recipient-site complications, however, showed that hematoma and seroma occurred significantly more often in immediate reconstructions, while wound problems were more frequently observed in delayed reconstructions. Significant differences were still found after correction for potential confounders, except for wound problems. Adequate hemostasis and prevention of seroma formation are particularly important in immediate breast reconstruction. Nevertheless, patients eligible for immediate reconstruction should be counseled regarding the safety of immediate DIEP flap breast reconstruction, thereby taking the improved aesthetic outcome and reduced psychological distress into account.

## Electronic supplementary material

Below is the link to the electronic supplementary material.

Supplementary material 1 (PDF 146 kb)
