# Supplementary material for: Complications following immediate compared to delayed deep inferior epigastric artery perforator flap breast reconstructions
Source: Breast Cancer Res Treat. 2018 Feb 5;169(2):349–57. doi: 10.1007/s10549-018-4695-0 (PMC5945748; doi:10.1007/s10549-018-4695-0)
Supplement: Supplementary file 1 — Supplementary material 1 (PDF 146 kb) [file 10549_2018_4695_MOESM1_ESM.pdf]

# Online Resource 1: Patient as unit of analysis

**Article title:**
Complications of immediate compared to delayed deep inferior epigastric artery perforator flap breast reconstructions

**Journal name:**
Breast Cancer Research and Treatment

**Author names:**
J. Beugels*[1,2], L. Bod[3], S.M.J. van Kuijk[4], S.M.H. Tuinder[1], S.S. Qiu[1], E.M. Heuts[3], A. Piatkowski[1,5], R.R.W.J. van der Hulst[1,5,6]

**Affiliations:**
[1)] Department of Plastic, Reconstructive and Hand Surgery, Maastricht University Medical Center, Maastricht, the Netherlands
[2)] GROW – School for Oncology and Developmental Biology, Maastricht University Medical Center, Maastricht, the Netherlands
[3)] Department of Surgery, Maastricht University Medical Center, Maastricht, the Netherlands
[4)] Department of Clinical Epidemiology and Medical Technology Assessment (KEMTA), Maastricht University Medical Center, Maastricht, the Netherlands
[5)] Department of Plastic, Reconstructive and Hand Surgery, VieCuri Medical Center, Venlo, the Netherlands
[6)] Department of Plastic, Reconstructive and Hand Surgery, Zuyderland Medical Center, Sittard-Geleen, the Netherlands

*** Corresponding author:**
Jop Beugels, *M.D.*
Department of Plastic, Reconstructive and Hand Surgery, Maastricht University Medical Centre, P.O. Box 5800, 6202, AZ, Maastricht, the Netherlands. (+31) 43 387 7481; jop.beugels@mumc.nl

**Table 1. Recipient-site complications (*n* = 737 patients)**

| | Timing of reconstruction | | | | | |
|---|---|---|---|---|---|---|
| | Immediate (*n* = 291) *n* (%) | Delayed (*n* = 446) *n* (%) | OR (95% CI) | *p* value | Adjusted OR (95% CI) [a] | Adjusted *p* value [a] |
| Major complication (≥1) | 33 (11.3) | 52 (11.7) | 0.97 (0.61-1.54) | 0.895 | 0.77 (0.47-1.29) | 0.320 |
| Total flap loss | 6 (2.1) | 13 (2.9) | 0.70 (0.26-1.87) | 0.477 | 0.46 (0.20-1.10) | 0.081 |
| Partial flap loss | 13 (4.5) | 25 (5.6) | 0.79 (0.40-1.57) | 0.496 | 0.72 (0.33-1.58) | 0.415 |
| Venous congestion | 18 (6.2) | 21 (4.7) | 1.33 (0.70-2.55) | 0.383 | 0.93 (0.47-1.85) | 0.842 |
| | | | | | | |
| Minor complication (≥1) | 83 (28.5) | 121 (27.1) | 1.07 (0.77-1.49) | 0.680 | 1.11 (0.78-1.59) | 0.552 |
| Infection | 23 (7.9) | 37 (8.3) | 0.95 (0.55-1.63) | 0.849 | 1.03 (0.57-1.87) | 0.922 |
| Hematoma | 34 (11.7) | 17 (3.8) | 3.34 (1.83-6.10) | <0.001 | 3.03 (1.60-5.73) | 0.001 |
| Seroma | 11 (3.8) | 4 (0.9) | 4.34 (1.37-13.8) | 0.013 | 4.92 (1.24-19.6) | 0.024 |
| Fat necrosis | 36 (12.4) | 63 (14.1) | 0.86 (0.55-1.33) | 0.495 | 0.87 (0.55-1.38) | 0.554 |
| Wound problems | 31 (10.7) | 77 (17.3) | 0.57 (0.37-0.89) | 0.014 | 0.62 (0.39-0.98) | 0.041 |

[a] Adjusted for unilateral vs. bilateral reconstruction, body mass index (kg/m$^2$), smoking status (yes vs. no), radiation therapy (yes vs. no), chemotherapy (yes vs. no), endocrine therapy (yes vs. no), and university vs. community hospital.

**Table 2. Subgroup analysis recipient-site complications of breast cancer patients only (*n* = 664 patients)**

| | Timing of reconstruction | | | | | |
|---|---|---|---|---|---|---|
| | Immediate (*n* = 234) *n* (%) | Delayed (*n* = 430) *n* (%) | OR (95% CI) | *p* value | Adjusted OR (95% CI) [a] | Adjusted *p* value [a] |
| Major complication (≥1) | 21(9.0) | 48 (11.2) | 0.79 (0.46-1.35) | 0.378 | 0.73 (0.41-1.30) | 0.279 |
| Total flap loss | 2 (0.9) | 12 (2.8) | 0.30 (0.07-1.35) | 0.117 | 0.25 (0.06-1.16) | 0.076 |
| Partial flap loss | 8 (3.4) | 24 (5.6) | 0.60 (0.27-1.36) | 0.218 | 0.56 (0.22-1.41) | 0.219 |
| Venous congestion | 12 (5.1) | 19 (4.4) | 1.17 (0.56-2.45) | 0.679 | 1.07 (0.51-2.23) | 0.865 |
| | | | | | | |
| Minor complication (≥1) | 65 (27.8) | 114 (26.5) | 1.07 (0.75-1.52) | 0.725 | 1.19 (0.81-1.73) | 0.375 |
| Infection | 19 (8.1) | 36 (8.4) | 0.97 (0.54-1.73) | 0.910 | 1.09 (0.59-2.02) | 0.786 |
| Hematoma | 29 (12.4) | 14 (3.3) | 4.20 (2.17-8.13) | <0.001 | 3.97 (2.03-7.76) | <0.001 |
| Seroma | 7 (3.0) | 3 (0.7) | 4.39 (1.12-17.1) | 0.033 | 7.97 (1.93-32.8) | 0.004 |
| Fat necrosis | 28 (12.0) | 61 (14.2) | 0.82 (0.51-1.33) | 0.423 | 0.87 (0.53-1.43) | 0.594 |
| Wound problems | 21 (9.0) | 74 (17.2) | 0.47 (0.28-0.79) | 0.004 | 0.55 (0.33-0.93) | 0.025 |

[a] Adjusted for unilateral vs. bilateral reconstruction, body mass index (kg/m$^2$), smoking status (yes vs. no), radiation therapy (yes vs. no), chemotherapy (yes vs. no), endocrine therapy (yes vs. no), and university vs. community hospital.

**Table 3. Flap re-explorations (*n* = 737 patients)**

| | Timing of reconstruction | | |
|---|---|---|---|
| | Immediate (*n* = 291) *n* (%) | Delayed (*n* = 446) *n* (%) | *p* value |
| Re-exploration | 28 (9.6) | 37 (8.3) | 0.535 |
| Reanastomosis | 19 (6.5) | 20 (4.5) | 0.228 |
| Reason re-exploration | | | |
| Arterial insufficiency | 4 (1.4) | 11 (2.5) | 0.305 |
| Venous insufficiency | 19 (6.5) | 20 (4.5) | 0.225 |
| Hematoma | 7 (2.4) | 6 (1.3) | 0.285 |
| Kinking | 4 (1.4) | 5 (1.1) | 0.745 [a] |
| Infection | 0 (0) | 1 (0.2) | 1.000 [a] |
| Other | 1 (0.3) | 2 (0.4) | 1.000 [a] |
| Result re-exploration [b] | | | |
| Viable flap | 20 (71.5) | 21 (56.8) | 0.225 |
| Partial flap loss | 2 (7.1) | 8 (21.6) | 0.167 [a] |
| Total flap loss | 6 (21.4) | 8 (21.6) | 0.985 |

[a] Fisher's Exact test was used.

[b] As a percentage of the total flaps that required re-exploration (immediate group: *n* = 28; delayed group: *n* = 37).
